# Application of machine learning algorithms to predict and assess factors related to internal carotid siphon aneurysm occlusion treated with flow diversion

**DOI:** 10.1007/s00234-026-03982-1

**Published:** 2026-03-25

**Authors:** Hongbin Li, Shaofeng Shui, Ji Ma, Tengfei Li, Dong Guo

**Affiliations:** First Affiliated Hospital of Zhengzhou University, Zhengzhou, China

**Keywords:** Internal carotid siphon aneurysm, Endovascular treatment, Flow diversion, Machine learning algorithms

## Abstract

**Purpose:**

Numerous clinical indicators have been identified as risk factors for aneurysm occlusion and used to development of various Flow Diversion Predictive Score grading scales. The objective of this study is to predict and assess factors related to Internal carotid siphon aneurysm occlusion, by leveraging Machine Learning(ML) Algorithms.

**Methods:**

We conducted a retrospective analysis of patients treated with flow diversion from January 2020 to December 2023 in our department. The samples were randomly divided into derivation and validation groups in a 70/30 ratio. We employed six ML algorithms, including LR, DT, SVM, RF, GBM, and XGBoost, these algorithms were utilized alongside preoperative and intraoperative clinicopathological characteristics to develop predictive models.

**Results:**

A total of 297 patients with 342 target aneurysms were included. Multivariate logistic regression analyses identified aneurysm orientation, W, BNF, IA, incorporated branch vessels, and adjunct coil deployment as independent predictors. Additionally, a random forest feature selection analysis was conducted to identify potentially significant factors based on importance scores, IA performed the most outstandingly. Combining with previously reported literature, we ultimately determined the variables for inclusion in six ML models: IA, BNF, incorporated branch vessels, adjunct coil deployment, ostium area, CND, NR, and Dmax. Among these models, the GBM showed superior performance with an AUROC of 0.766.

**Conclusion:**

By integrating preoperative and intraoperative factors, ML algorithms can achieve acceptable predictions. If widely implemented, this approach could serve as a valuable reference for selecting surgical methods for specific aneurysms prior to operation in clinical practice.

**Supplementary Information:**

The online version contains supplementary material available at 10.1007/s00234-026-03982-1.

## Introduction

The introduction of flow diversion devices(FD) has fundamentally transformed the paradigm of endovascular treatment for intracranial aneurysms, transitioning from traditional intra-aneurysmal coiling to a focus on blood flow diversion at the neck of the aneurysm. This approach has gained increasing acceptance with the widespread implementation of FD represented by Pipeline Embolization Devices (PED, Covidien/Medtronic) [1–5]. Numerous comparative studies have demonstrated that FD can achieve a significantly higher rate of complete aneurysm occlusion compared to stent-assisted coiling, particularly in cases involving large, giant, and other complex aneurysms [6], the overall 1-year complete occlusion rate can reach as high as 85.5%. Consequently, FD has emerged as one of the primary modalities for treating intracranial aneurysms.

The siphon segment of the internal carotid artery is a critical site for intracranial aneurysms, accounting for 34.1% to 46.7% of cases [7–9], aneurysms located at this site exhibit a diverse array of characteristics based on the anatomy of the siphon segment, including variations in location, size, shape, neck configuration, protrusion direction, and blood flow injection angle. Traditional endovascular treatments may encounter significant technical challenges and risks; these include difficulties in catheter insertion or stabilization due to angulation, inadequate occlusion of the aneurysm neck, and an elevated risk of intraoperative rupture as well as complications such as spring coil detachment and postoperative recurrence. Both surgical clipping and endovascular interventions are associated with high complication rates, the 30-day overall morbidity and mortality are 13.7% and 9.3% respectively [7]. Specifically, large intracranial aneurysms treated with conventional stent assistance or spring coil embolization demonstrate recurrence rates ranging from 32.5% to 44%, alongside re-operation rates between 26% and 40% [10–13]. 

FD offers clinicians additional strategies for managing these complex aneurysms [14]. However, it is important to acknowledge that procedure-related morbidity and mortality remain non-negligible concerns [15]. Moreover, certain aneurysms may fail to close adequately or may experience delayed closure with potential risks for enlargement or rupture thereafter [16]. Numerous studies have investigated various predictive factors of aneurysm occlusion(such as preoperative clinical factors, preoperative and postoperative imaging morphological features of both the aneurysm and the parent artery, operative style, etc.) [17–20] Some investigations have even proposed clinical scoring scales aimed at predicting aneurysm occlusion based on these identified factors [21–23], nevertheless, predictive factors remain ambiguous and contentious within the literature while discrepancies among different scoring scales persist without achieving widespread consensus.

The internal carotid siphon aneurysm, as a primary target for FD, has not been subjected to a detailed analysis of its predictors for aneurysm occlusion. Machine learning (ML), as a novel type of artificial intelligence, is starting to be widely applied to health-care data analysis [24]. By leveraging the robust predictive capabilities of ML algorithms, it may be feasible to identify potential predictors that can surpass traditional statistical modeling in certain instances, thereby enhancing the prediction accuracy for aneurysm occlusion. Consequently, this study aims to develop ML-based models utilizing preoperative and intraoperative clinicopathological characteristics to predict and assess the likelihood of occlusion in internal carotid siphon aneurysms treated with FD.

## Methods

### Patients

We conducted a retrospective analysis of patients who underwent endovascular treatment for internal carotid siphon aneurysms utilizing flow diversion techniques in our department from January 2020 to December 2023. Exclusion criteria included non-internal carotid artery siphon segment aneurysms (C4-C7), dissecting aneurysms, fusiform aneurysms, false aneurysms, those with subarachnoid hemorrhage or prior interventions, target aneurysm maximum diameters < 3 mm, perioperative mortality, loss to follow-up, and unavailability of DSA imaging.

### Data collection

We conducted a systematic review of patient demographics, including gender, age, hypertension, hyperlipidemia, and diabetes; as well as the clinicopathological variables associated with aneurysms: internal carotid artery side (left/right), location (C4/C5/C6/C7), orientation (concave/convex/flank), maximum diameter (Dmax), height (H), width (W), clinical neck diameter (CND), parent artery diameter (PAD), neck ratio (NR), height-to-width ratio (H/W), aspect ratio (AR), bottle-neck factor (BNF), shear rate (SR), aneurysm volume, ostium area, volume-to-ostium area ratio (VOR), inflow angle (IA) and incorporated branch vessels along with adjunct coil deployment.

### Postoperative follow-up evaluation

In clinical practice, the standard follow-up protocol is delineated as follows: The initial angiographic assessment occurs 3 to 6 months post-FD implantation. For patients exhibiting complete aneurysm occlusion during the first DSA follow-up, further imaging and evaluations are not routinely mandated; conversely, for those without complete occlusion, assessments should be conducted every 6 to 12 months. However, adherence to these guidelines may vary among patients, resulting in incomplete follow-up data that precludes definitive evaluation of aneurysm occlusion within the specified timeframe. Consequently, we propose that if DSA demonstrates aneurysm occlusion within 6 months, it can be classified as occluded at that time point. Conversely, if no occlusion is observed after a 6-month period of monitoring, it will be categorized as non-occluded; cases lacking sufficient evaluability will be excluded from this study. The status of aneurysm occlusion is assessed utilizing the O’Kelly-Marotta (OKM) grading scale—an established framework specifically designed for evaluating intracranial aneurysms treated via flow diversion techniques. The OKM grading scale classifies filling status into four categories: A (complete > 95%), B (incomplete 5%–95%), C (neck remnant < 5%), and D (no filling = 0%). Notably, an OKM grade of D signifies complete occlusion [25]. To ensure the consistency of the data, aneurysm clinicopathologic variables were jointly measured and aneurysm occlusion status was jointly determined by the two specific interventionalist.

### Statistical analysis

Patient characteristics were summarized using percentages and mean ± SD ranges as appropriate. Categorical variables were analyzed with Fisher’s exact test or Pearson’s chi-square test, while continuous variables between groups were compared utilizing the Mann-Whitney U test or Student’s t-test. To identify potentially significant predictors, both univariate and multivariate logistic regression analyses were conducted to compute odds ratios (ORs) along with 95% confidence intervals (CIs). Furthermore, a random forest approach was employed for feature selection analysis to determine significant factors based on their importance scores; these findings, in conjunction with existing literature, informed the final selection of variables incorporated into the ML models.

Employ the 10 events per variable rule to estimate and ensure that the sample size included in the prediction model is sufficient. In this study, we randomly divided our dataset into a derivation set (70%) for the development of the ML models and a validation set (30%) for performance evaluation. It is essential that the samples are equally distributed across both sets. We developed six types of ML algorithms to model our data: Logistic Regression (LR), Decision Tree (DT), Support Vector Machine(SVM), Random Forest (RF), Gradient Boosting Machine (GBM), and Extreme Gradient Boosting (XGBoost). During the derivation process, hyperparameter tuning was implemented for ML-based models to mitigate overfitting, with 5-fold cross-validation employed as the optimal method for hyperparameter selection. Subsequently, these ML algorithms were further trained using R software to predict the risk of aneurysm occlusion. We assessed the predictive performance of each ML classifier on validation sets by calculating metrics such as the area under the receiver operating characteristic curve (AUROC), specificity, and overall accuracy. In comparing the performance of different ML algorithms, a higher AUROC value—closer to 1—indicated superior classification capability. Additionally, we presented a relative importance ranking of factors influencing aneurysm occlusion prediction.

## Results

### Patient characteristics

A total of 409 patient records were collected, among which 42 patients were lost to follow-up postoperatively (including 3 patients who were found deceased during the postoperative period or follow-up). Additionally, in 70 cases, it was not possible to ascertain whether the aneurysm had occluded at the 6-month follow-up due to adherence issues with follow-up protocols. Ultimately, a cohort of 297 patients with 342 target internal carotid artery siphon aneurysms was enrolled and confirmed; the incidence of aneurysm occlusion at 6 months post-follow-up was found to be 57.31%. Demographic and clinicopathological variables for the entire cohort were recorded and compared based on their aneurysm occlusion status within 6 months (Table 1).

**Table 1:** Demographic and clinicopathologic variables of the whole cohort grouped by aneurysm occlusion status within 6 months

Charteristics		Total (*N* = 342) No (%)	Residual (*N* = 146) No (%)			Occlusion (*N* = 196) No (%)	*p*
Gender							0.812
Male	259 (75.73)		112 (76.71)			147 (75.00)	
Female	83 (24.27)		34 (23.29)			49 (25.00)	
Age(years)	55.67 ± 10.06		56.33 ± 9.01			55.17 ± 10.77	0.292
Hypertension							0.142
No	204 (59.65)		80 (54.79)			124 (63.27)	
Yes	138 (40.35)		66 (45.21)			72 (36.73)	
Hyperlipidemia							1.000
No	334 (97.66)		143 (97.95)			191 (97.45)	
Yes	8 (2.34)		3 (2.05)			5 (2.55)	
Diabetes							0.221
No	313 (91.52)		130 (89.04)			183 (93.37)	
Yes	29 (8.48)		16 (10.96)			13 (6.63)	
Internal carotid side							0.607
Left	208 (60.82)		86 (58.90)			122 (62.24)	
Right	134 (39.18)		60 (41.10)			74 (37.76)	
Location							0.054
C4	16 (4.68)		8 (5.48)			8 (4.08)	
C5	10 (2.92)		1 (0.68)			9 (4.59)	
C6	201 (58.77)		80 (54.79)			121 (61.73)	
C7	115 (33.63)		57 (39.04)			58 (29.59)	
Aneurysm orientation							< 0.001
Concave	92 (26.90)		28 (19.18)			64 (32.65)	
Convex	87 (25.44)		56 (38.36)			31 (15.82)	
Flank	163 (47.66)		62 (42.47)			101 (51.53)	
Dmax(mm)	6.50 ± 4.69		6.99 ± 5.76			6.13 ± 3.66	0.092
H(mm)	5.17 ± 3.88		5.69 ± 4.87			4.78 ± 2.90	0.032
W(mm)	5.31 ± 3.99		5.821 ± 5.03			4.93 ± 2.96	0.041
CND(mm)	3.83 ± 1.60		4.26 ± 1.87			3.52 ± 1.28	< 0.001
PAD(mm)	3.87 ± 0.56		3.88 ± 0.56			3.87 ± 0.55	0.871
NR	0.99 ± 0.38		1.10 ± 0.44			0.91 ± 0.30	< 0.001
AR	1.32 ± 0.56		1.27 ± 0.56			1.36 ± 0.56	0.152
H/W	1.03 ± 0.33		1.04 ± 0.30			1.02 ± 0.35	0.657
BNF	1.35 ± 0.59		1.26 ± 0.51			1.41 ± 0.63	0.018
SR	1.33 ± 0.99		1.46 ± 1.25			1.24 ± 0.72	0.039
Aneurysm volume(mm^3^)	346.66 ± 1496.98		575.98 ± 2204.12			175.84 ± 487.49	0.014
Ostium area(mm^2^)	13.53 ± 14.46		16.93 ± 19.24			10.99 ± 8.68	< 0.001
VOR	13.09 ± 33.21		14.77 ± 44.13			11.83 ± 21.84	0.419
IA	84.34 ± 26.61		92.90 ± 25.49			77.95 ± 25.67	< 0.001
Incorporated branch vessels							0.004
No	282 (82.46)			110 (75.34)	172 (87.76)		
Yes	60 (17.54)			36 (24.66)	24 (12.24)		
Adjunct coil deployment							0.005
No	267 (78.07)			125 (85.62)	142 (72.45)		
Yes	75 (21.93)			21 (14.38)	54 (27.55)		

Dmax, maximum diameter; H, height; W, width; CND, clinical neck diameter; PAD, parent artery diameter; NR, neck ratio; H/W, height-to-width ratio; AR, aspect ratio; BNF, bottle-neck factor; SR, shear rate; VOR, volume-to-ostium area ratio; IA, inflow angle

### Variable selection for ML algorithms

As illustrated in Table 2, the univariable analysis revealed that aneurysm orientation, H, W, CND, NR, BNF, SR, aneurysm volume, ostium area, IA, incorporated branch vessels, and adjunct coil deployment were all significantly associated with aneurysm occlusion (*P* < 0.05). In the subsequent multivariable logistic regression analysis incorporating these significant variables, results indicated that aneurysm orientation(OR 0.287, 95%CI 0.133–0.604), W(OR 0.446, 95%CI 0.236–0.802), BNF(OR 66.714, 95%CI 5.622-1067.655), IA(OR 0.978, 95%CI 0.967–0.988), incorporated branch vessels(OR 0.423, 95%CI 0.210–0.834) and adjunct coil deployment(OR 8.438, 95%CI 3.260-24.418) were independent influence factors of aneurysm occlusion.

**Table 2:** Univariate and multivariate logistic regression analysis of variables in predicting aneurysm occlusion status in whole cohort

Charteristics	Univariate analysis		Multivariate analysis	
	OR (95%CI)	*p*	OR (95%CI)	*p*
Gender	1.098(0.667–1.823)	0.715		
Age	0.988(0.967–1.010)	0.292		
Hypertension	0.704(0.454–1.089)	0.115		
Hyperlipidemia	1.248(0.301–6.163)	0.764		
Diabetes	0.577(0.264–1.239)	0.159		
Internal carotid side(L/R)	0.869(0.561–1.349)	0.531		
Location				
C4	Reference			
C5	9.000(1.247-186.647)	0.060		
C6	1.513(0.536–4.270)	0.427		
C7	1.018(0.352–2.944)	0.974		
Aneurysm orientation				
Concave	Reference		Reference	
Convex	0.242(0.128–0.448)	<0.001	0.287(0.133–0.604)	0.001
Flank	0.713(0.410–1.222)	0.223	0.733(0.377–1.402)	0.352
Dmax	0.961(0.916–1.222)	0.098		
H	0.940(0.883–0.995)	0.038	0.751(0.372–1.446)	0.349
W	0.945(0.892–0.998)	0.047	0.446(0.236–0.802)	0.009
CND	0.727(0.618–0.844)	<0.001	1.819(0.414–7.785)	0.391
PAD	0.968(0.658–1.428)	0.871		
NR	0.234(0.117–0.443	<0.001	0.085(0.001–4.209)	0.212
AR	1.343(0.904–2.045)	0.155		
H/W	0.861(0.445–1.668)	0.656		
BNF	1.626(1.094–2.517)	0.022	66.714(5.622-1067.655)	0.002
SR	0.783(0.603–0.985)	0.049	3.929(0.335–67.928)	0.257
Aneurysm volume	0.999(0.999–0.999)	0.033	1.000(0.999–1.001)	0.960
Ostium area	0.961(0.939–0.981)	<0.001	1.053(0.923–1.154)	0.332
VOR	0.997(0.990–1.004)	0.429		
IA	0.978(0.969–0.986)	<0.001	0.978(0.967–0.988)	<0.001
Incorporated branch vessels	0.426(0.239–0.749)	0.003	0.423(0.210–0.834)	0.014
Adjunct coil deployment	2.264(1.312–4.027)	0.004	8.438(3.260-24.418)	<0.001

Dmax, maximum diameter; H, height; W, width; CND, clinical neck diameter; PAD, parent artery diameter; NR, neck ratio; H/W, height-to-width ratio; AR, aspect ratio; BNF, bottle-neck factor; SR, shear rate; VOR, volume-to-ostium area ratio; IA, inflow angle

**Fig. 1:**
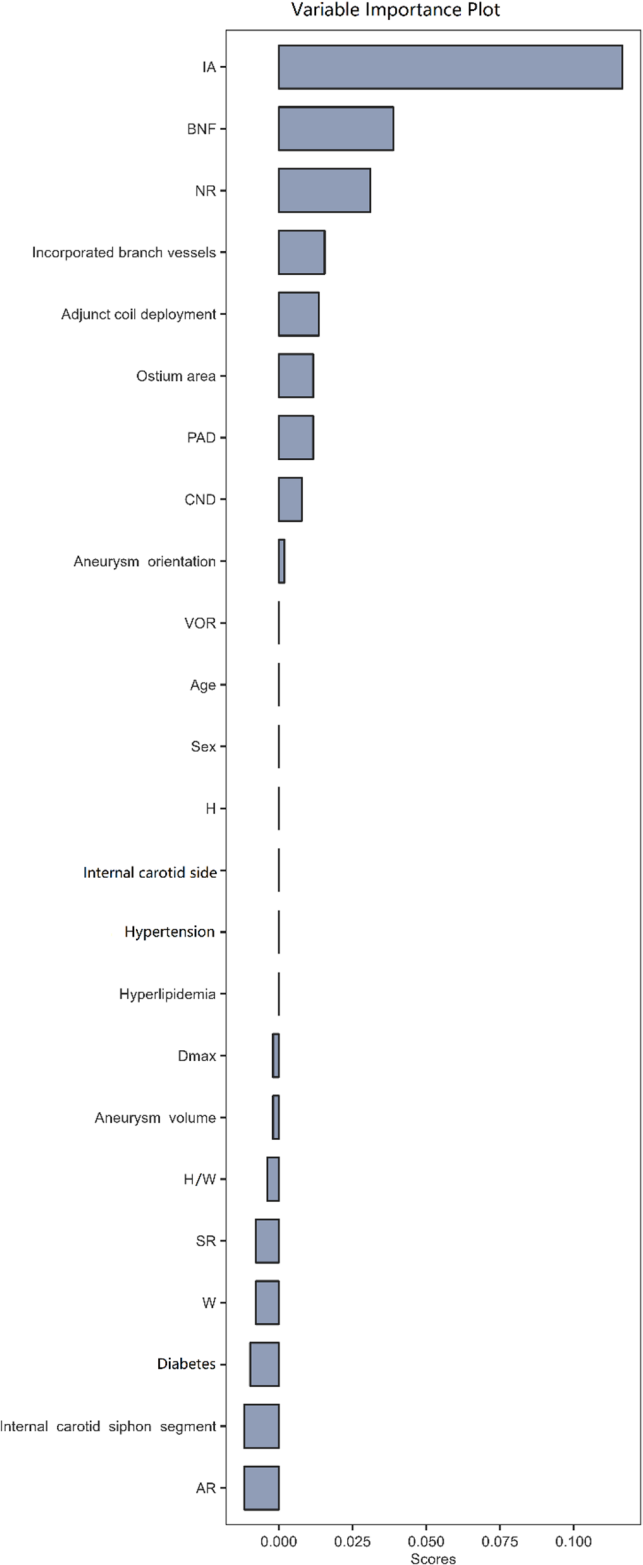
Random forest was employed for feature selection analysis to determine significant variables based on their importance scores. Note: IA, inflow angle; BNF, bottle-neck factor; NR, neck ratio; PAD, parent artery diameter; CND, clinical neck diameter; VOR, volume-to-ostium area ratio; H, height; Dmax, maximum diameter; H/W, height-to-width ratio; SR, shear rate; W, width; AR, aspect ratio

Furthermore, a random forest was used for feature selection analysis, and the most significant factors were selected based on their importance scores (Fig. 1). Combining these findings with previously reported literature, the final variables included in the ML model were IA, BNF, incorporated branch vessels, adjunct coil deployment, ostium area, CND, NR, and Dmax.

### Performance of ML algorithms

**Fig. 2:**
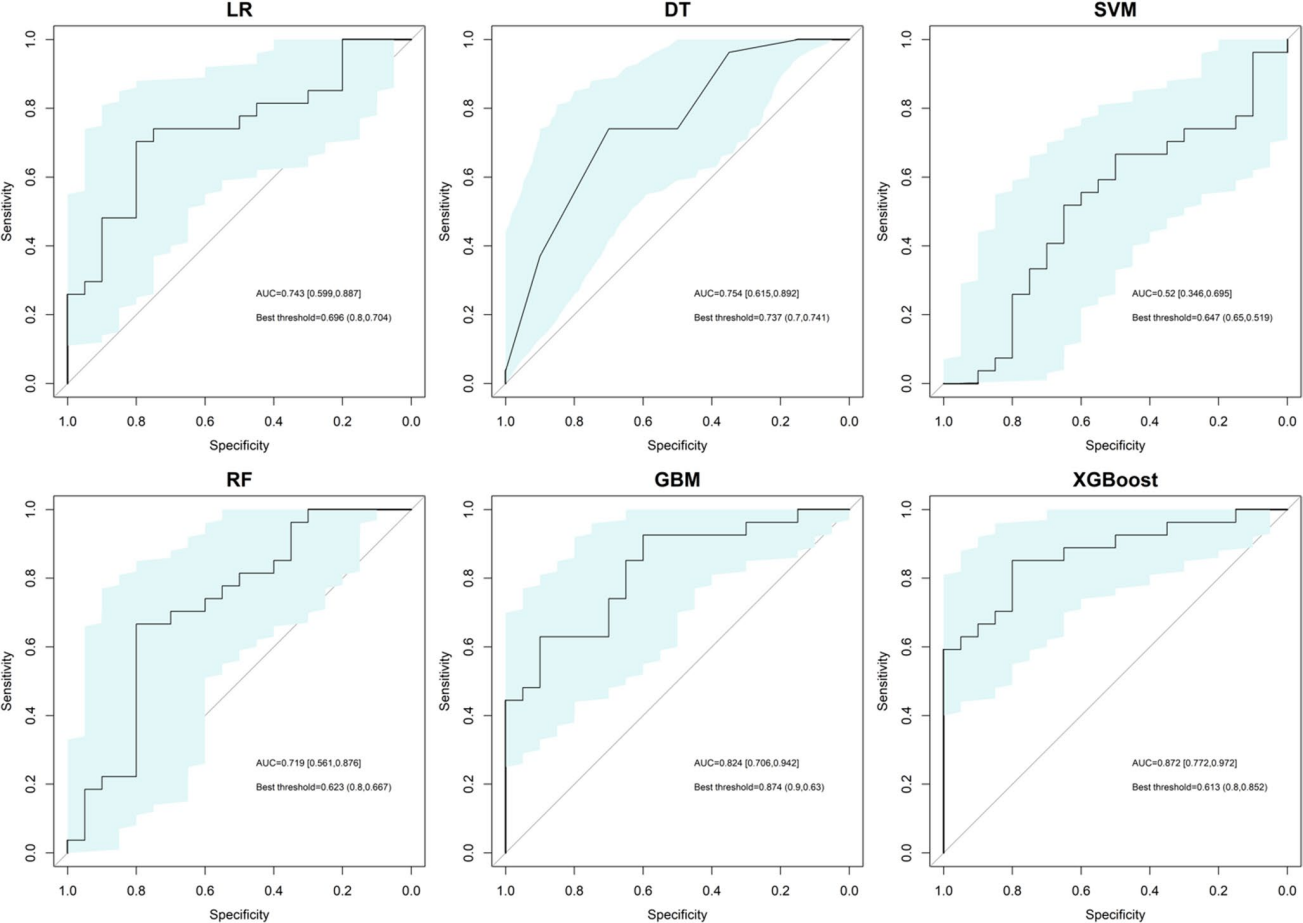
The AUROC curves of each ML algorithm for prediction of aneurysm occlusion in the derivation set

Of these samples,70% (239/342) were allocated to the derivation set while 30% (103/342) comprised the validation set; baseline characteristics are comprehensively outlined in the supplementary materials. The AUROC curves for each model in the derivation set are illustrated in Fig. 2, where the XGBoost algorithm exhibits the highest area under the AUROC value of 0.872, followed by the GBM of 0.824, DT of 0.754, LR of 0.743, RF of 0.719, and SVM of 0.520; this suggests that both XGBoost and GBM algorithms demonstrate superior performance in the derivation set. In contrast, when evaluating models on the validation set, GBM achieves the highest AUROC of 0.766 alongside an F1-Score of 0.807 and an Accuracy of 0.766; indicating that GBM outperforms all other algorithms in this context. The comprehensive results for all models are presented in Table 3.

**Table 3:** Predictive performance comparison of the six types of machine learning algorithms in the validation sets

Model Name	Accuracy	Prevalence	Recall	F1-Score	MCC	AUROC	Presicion	Specificity	FNR	FPR
LR	0.617	0.574	0.815	0.710	0.187	0.606	0.629	0.350	0.185	0.650
DT	0.638	0.574	0.741	0.702	0.248	0.627	0.667	0.500	0.259	0.500
SVM	0.553	0.574	0.815	0.677	0.019	0.512	0.579	0.200	0.185	0.800
RF	0.660	0.574	0.778	0.724	0.290	0.651	0.677	0.500	0.222	0.500
GBM	0.766	0.574	0.852	0.807	0.516	0.766	0.767	0.650	0.148	0.350
XGBoost	0.745	0.574	0.889	0.800	0.474	0.756	0.727	0.550	0.111	0.450

LR, Logistic regression; DT, Decision tree; SVM, Support Vector Machine; RF, Random forest; GBM, Gradient boosting machine; XGBoost, Extreme gradient boosting

### Relative Importance of variables in ML algorithms

**Fig. 3:**
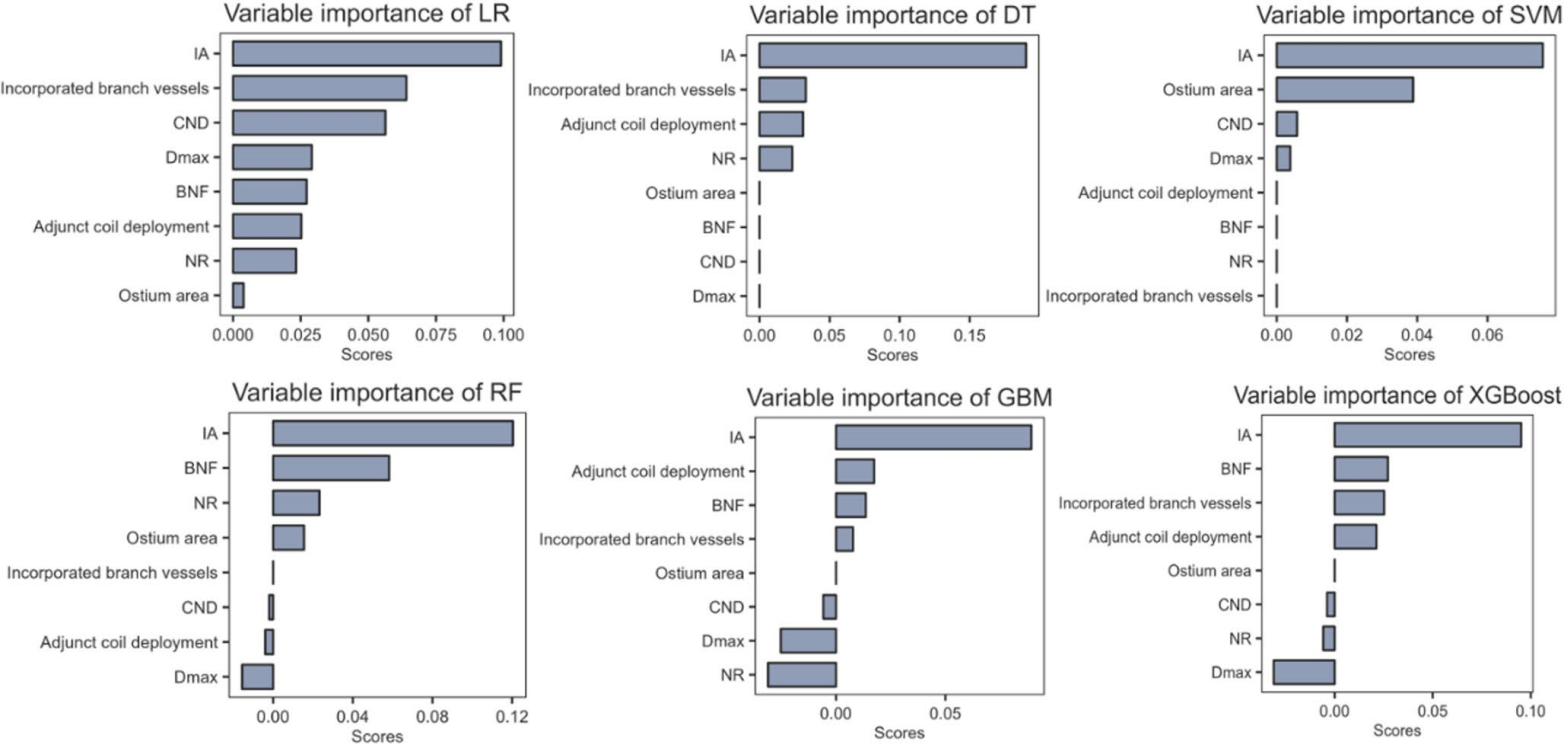
The relative importance of variables in each ML algorithm predicting aneurysm occlusion is illustrated, the factor of IA consistently ranks at the top. Note: IA, inflow angle; CND, clinical neck diameter; Dmax, maximum diameter; BNF, bottle-neck factor; NR, neck ratio

The relative importance of variables in each ML algorithm predicting aneurysm occlusion is illustrated in Fig. 3. Notably, while there are significant differences in the variable importance across these algorithms, the factor of IA consistently ranks at the top. In the GBM model, the high-ranking variables are arranged in descending order as follows: IA, NR, Dmax, adjunct coil deployment, BNF, incorporated branch vessels, CND, ostium area.

## Discussion

Endovascular flow diversion therapy differs from embolization therapy, which necessitates a specific duration for the formation of new endothelium on the stent surface at the aneurysm neck to achieve occlusion. This approach is not without limitations; not all aneurysms can be occluded or do so within a short timeframe. It is primarily suitable for certain types of aneurysms, underscoring the importance of selecting appropriate candidates based on clinical characteristics to facilitate more rapid and effective aneurysm occlusion.

A retrospective angiographic follow-up analysis of 967 aneurysms from the largest multi-center PED series in China revealed that female, hyperlipidemia, vertebral aneurysms, PED plus coiling, and blood flow detained to venous phase were significant predictors of aneurysm occlusion in multivariate analysis. Conversely, factors such as age, history of SAH, > 6 months dual antiplatelet therapy post-PED implant, aneurysm maximal diameter, and fusiform morphology were associated with incomplete occlusion. For the posterior circulation cohort, there were no variables to compare with [20]. The largest single-center clinical study from Johns Hopkins University School of Medicine conducted a retrospective analysis of 491 cases of PED treatment for anterior circulation aneurysms. Multivariate analysis revealed that at 6 months, advancing age, increasing aneurysm size, history of SAH and adjunctive coiling were significant predictors of aneurysm occlusion; whereas at 12 months, the predictors included male sex, increasing aneurysm size, branch vessel inclusion within the treated aneurysm, along with adjunctive coiling [19]. Another retrospective analysis conducted across 3 experienced vascular centers examined 316 aneurysms treated with PED. Multivariable analysis revealed that older age (> 70 years), greater maximal diameter (≥ 15 mm), and fusiform morphology were strong independently associated with higher rates of incomplete occlusion at last follow-up [18]. In addition to the aforementioned factors, clinical and pathological variables such as location (C4/C5/C6/C7), orientation (concave/convex/flank), H, W, CND, PAD, NR, H/W, AR, BNF, SR, aneurysm volume, ostium area, VOR, and IA have also been incorporated in prior studies to explore their potential association with aneurysm occlusion [17, 20, 26–29]. It should not be overlooked that the device itself is also an important influencing factor. The respective comparative sizes of the device and the recipient artery, the curvature of the device, as well as the method of deploying the device will affect the porosity of the device, thereby influencing the hemodynamics of the carotid artery carrying the aneurysm, and ultimately affecting aneurysm occlusion, especially in curved vessels such as the carotid siphon [30]. 

Review of the existing prediction scoring models(FDSS、ABC、DIANES、4 F-FPS) reveals significant variability in the selected variables among them. The Flow Diversion Stent Score (FDSS) is a straightforward outcomes-based scale to characterize results after FD treatment; it assigns points based on age ≥ 60 years (1 point), aneurysm size ≥ 15 mm (1 point), presence of side branches (1 point), and post-treatment Raymond score (1–3 points). Patients with FDSS ≥ 4 were more likely to have residual aneurysm filling on follow-up angiography [21]. The ABC scoring system consisted of: Age (< 60 years old: 0, 60–69 years: 1, 70–79: 2, and ≥ 80: 3), Branch coming out of the aneurysm dome/neck (yes: 2, no: 0), and Cigarette smoking history (never smoker: 1, current or past smoker: 0). A value ≥ 2 proved to be reliable in predicting the risk of incomplete occlusion [31]. The DIANES score employs logistic regression and ML via CART algorithms for feature selection, ultimately 6 significant variables (diameter, indication, parent artery diameter ratio, neck ratio, side-branch artery, and sex) were identified from a pool of 30 pre-treatment features and incorporated into the ML model, demonstrating robust predictive performance. Nevertheless, the study encompasses a limited number of cases, and the scoring parameters are intricate and challenging to remember [23]. In contrast, The Flow Diversion Predictive Score (4 F-FPS) builds upon previously established grading scales (OKM, Kamran, SMART) [25, 32, 33] for angiographic outcomes and identifies 4 critical factors influencing aneurysm occlusion, denoted by the acronym ‘F’: fusiform shape, flow-jet, filling, and final stasis. The scoring system aggregates the scores of these 4 factors, yielding a total score that ranges from 0 to 5. A score of ≥ 3 correlates with an estimated aneurysm occlusion rate of at least 78% [22]. 

The carotid internal artery siphon segment is not only a high-risk site for aneurysms, but also the main implantation site for blood flow diversion devices. The anatomy of aneurysms at this site is diverse, and the blood flow dynamics are complex [34, 35]. The factors that predict aneurysm closure have not been specifically analyzed. The 4 F-FPS scoring system is excellent, but most of the included factors are angiographic results after FD implantation and can only be used for postoperative prediction. It cannot be used for preoperative assessment of aneurysm occlusion rate, which may not be available for clinical daily practice to provide a reference for the choice of surgical method for a certain aneurysm before operation. ML algorithm shows great predictive power, which distinguishes itself from linear models adopted by previous researches, however, little research in the available literature on applying it to prediction of aneurysm occlusion treated with FD [36, 37]. Based on the above, we conducted further discussions, which is what makes this study different and unique.

This single-center study reviewed data from 342 internal carotid artery siphon aneurysms and conducted univariate and multivariate analyses of the pre-operative characteristic variables, which showed that aneurysm orientation, W, BNF, IA, incorporated branch vessels and adjunct coil deployment were independent influence factors. Meanwhile employing random forest for feature selection analysis facilitates the identification of potentially significant factors. By integrating these findings with previously reported literature, we determined the final variables to be incorporated into the ML model: IA, BNF, incorporated branch vessels, adjunct coil deployment, ostium area, CND, NR, and Dmax.

In the ML model applied to the derivation set, the XGBoost algorithm achieved a superior AUROC value of 0.872, followed by GBM at 0.824 and DT at 0.754. This suggests that both XGBoost and GBM algorithms exhibit strong performance on the validation set. Conversely, in the validation set evaluation, The AUROC of GBM is the highest, which can comprehensively evaluate the discrimination ability of the classification model, and several other indicators such as Accuracy and Precision are also superior. This can preliminarily indicate that this model has advantages. The relative importance of variables offers a clear representation of their significance in predicting aneurysm occlusion across various ML algorithms. In descending order of importance within the GBM model, high-ranking variables are as follows: IA, NR, Dmax, adjunct coil deployment, BNF, incorporated branch vessels, CND, and ostium area; where IA, adjunct coil deployment, and BNF serve as positive predictors for aneurysm occlusion while the remaining variables act as negative predictors. These findings appear to align more closely with clinical practice and previous reports. Of course, given the differences in the variables included in the studies by Hammoud [36], as well as in the variable selection for ML and the model training process, the most important predictors also vary. Compared with traditional prediction methods, ML can reduce the dimensionality of a large number of variables, efficiently select the predictive factors with high contribution, and improve the prediction accuracy.

It is noteworthy that there are discernible trends in the evidence: the factor of IA consistently ranks at the top, although significant differences exist regarding the importance of variables among various ML algorithms. IA is recognized as a crucial discriminant for rupture status in sidewall-type aneurysms, computational fluid dynamic analysis showed that increasing IA leading to deeper migration of the flow recirculation zone into the aneurysm with higher peak flow velocities and a greater transmission of kinetic energy into the distal portion of the dome.[38] The association between IA and aneurysm occlusion treated with FD was first reported here; these findings advocate for incorporating IA into future studies. Should these predictive factors be validated across multi-center datasets, it may become feasible to develop specific predictive models and integrate them into user-friendly applications to help predict treatment outcomes in real time. This tool can assist interventionalists in choosing the most appropriate therapeutic modality tailored for each patient, based on clinical risk factors and the morphological characteristics of the aneurysm.

Nevertheless, this study is not without its limitations. Firstly, the nature of a retrospective study might have resulted in selection bias. Secondly, the ML algorithm model we developed was confined to one single institution, which might limit its generality, pending external validation. Lastly, due to the relatively small sample size and model consistency constraints, we did not pursue further development of a predictive scoring system.

## Conclusion

We developed and validated ML algorithms for individualized prediction of internal carotid siphon aneurysm occlusion treated with flow diversion by utilizing readily available preoperative and intraoperative factors. ML algorithms can achieve acceptable predictive capability for aneurysm occlusion with the GBM model exhibiting superior performance. Additionally, they are valuable for variable screening and ranking the relative importance of predictors. If widely implemented, this approach could serve as a valuable reference for selecting surgical methods for specific aneurysms prior to operation in clinical practice. 

## Supplementary Information

Below is the link to the electronic supplementary material.

Supplementary Material 1 (DOCX 20.4 KB)

## Data Availability

No datasets were generated or analysed during the current study.
