# Supplementary material for: Application of machine learning algorithms to predict and assess factors related to internal carotid siphon aneurysm occlusion treated with flow diversion
Source: Neuroradiology. 2026 Mar 25;68(4):1037–46. doi: 10.1007/s00234-026-03982-1 (PMC13139210; doi:10.1007/s00234-026-03982-1)
Supplement: Supplementary file 1 — Supplementary Material 1 (DOCX 20.4 KB) [file 234_2026_3982_MOESM1_ESM.docx]

Supplementary materials

Comparison of baseline characteristics between the derivation and validation sets.

| **Charteristics** | **Derivation set**  **(N=239) No (%)** | **Validation set**  **(N=103) No (%)** | **p** |
| --- | --- | --- | --- |
| **Gender** |  |  | 0.130 |
| Male | 187 (78.24) | 72 (69.90) |  |
| Female | 52 (21.76) | 31 (30.10) |  |
| **Age(years)** | 56.01±9.77 | 54.85±10.71 | 0.329 |
| **Hypertension** |  |  | 0.224 |
| No | 137 (57.32) | 67 (65.05) |  |
| Yes | 102 (42.68) | 36 (34.95) |  |
| **Hyperlipidemia** |  |  | 0.944 |
| No | 234 (97.91) | 100 (97.09) |  |
| Yes | 5 (2.09) | 3 (2.91) |  |
| **Diabetes** |  |  | 0.921 |
| No | 218 (91.21) | 95 (92.23) |  |
| Yes | 21 (8.79) | 8 (7.77) |  |
| **Internal carotid side** |  |  | 1 |
| Left | 145 (60.67) | 63 (61.17) |  |
| Right | 94 (39.33) | 40 (38.83) |  |
| **Location** |  |  | 0.41 |
| C4 | 11 (4.60) | 5 (4.85) |  |
| C5 | 5 (2.09) | 5 (4.85) |  |
| C6 | 132 (55.23) | 69 (66.99) |  |
| C7 | 91 (38.08) | 24 (23.30) |  |
| **Aneurysm orientation** |  |  | 0.970 |
| Concave | 65 (27.20) | 27 (26.21) |  |
| Convex | 60 (25.10) | 27 (26.21) |  |
| Flank | 114 (47.70) | 49 (47.57) |  |
| **Dmax**(mm) | 6.72±5.09 | 5.97±3.56 | 0.177 |
| **H**(mm) | 5.30±4.20 | 4.85±3.00 | 0.328 |
| **W**(mm) | 5.50±4.29 | 4.88±3.17 | 0.190 |
| **CND**(mm) | 3.93±1.75 | 3.61±1.14 | 0.095 |
| **PAD**(mm) | 3.87±0.55 | 3.86±0.58 | 0.862 |
| **NR** | 1.01±0.41 | 0.94±0.29 | 0.134 |
| **AR** | 1.33±0.57 | 1.32±0.51 | 0.953 |
| **H/W** | 1.02±0.31 | 1.06±0.35 | 0.280 |
| **BNF** | 1.36±0.61 | 1.32±0.54 | 0.503 |
| **SR** | 1.36±1.06 | 1.28±0.81 | 0.498 |
| **Aneurysm volume**(mm^3^) | 414.94±1749.95 | 188.23±556.88 | 0.199 |
| **Ostium area**(mm^2^) | 14.51±16.47 | 11.26±7.68 | 0.056 |
| **VOR** | 14.09±37.01 | 10.77±21.99 | 0.398 |
| **IA** | 84.16±26.33 | 84.74±27.35 | 0.855 |
| **Incorporated branch vessels** |  |  | 0.627 |
| No | 195 (81.59) | 87 (84.47) |  |
| Yes | 44 (18.41) | 16 (15.53) |  |
| **Adjunct coil deployment** |  |  | 0.244 |
| No | 182 (76.15) | 85 (82.52) |  |
| Yes | 57 (23.85) | 18 (17.48) |  |
| **Aneurysm occlusion status** |  |  | 0.911 |
| No | 103 (43.10) | 43 (41.75) |  |
| Yes | 136 (56.90) | 60 (58.25) |  |

Note: Dmax, maximum diameter; H, height; W, width; CND, clinical neck diameter; PAD, parent artery diameter; NR, neck ratio; H/W, height-to-width ratio; AR, aspect ratio; BNF, bottle-neck factor; SR, shear rate; VOR, volume-to-ostium area ratio; IA, inflow angle.
